# Effect on the Properties of Edible Starch-Based Films by the Incorporation of Additives: A Review

**DOI:** 10.3390/polym14101987

**Published:** 2022-05-13

**Authors:** Gurvendra Pal Singh, Sneh Punia Bangar, Tianxi Yang, Monica Trif, Vinod Kumar, Dinesh Kumar

**Affiliations:** 1 School of Bioengineering and Food Technology, Shoolini University of Biotechnology and Management Sciences, Bajhol, PO Sultanpur, Distt., Solan 173229, HP, India; gurvendranohwar@gmail.com; 2 Department of Food, Nutrition and Packaging Sciences, Clemson University, Clemson, SC 29634, USA; 3 Food, Nutrition and Health Program, Faculty of Land and Food System, The University of British Columbia, Vancouver, BC V6T 1Z4, Canada; tianxi.yang@ubc.ca; 4 Centre for Innovative Process Engineering (CENTIV) GmbH, Food Research Department, 28816 Stuhr, Germany; monica_trif@hotmail.com; 5 CENCIRA Agrofood Research and Innovation Centre, Ion Meșter 6, 400650 Cluj-Napoca, Romania; 6 Centre for Climate and Environmental Protection, School of Water, Energy and Environment, Cranfield University, Cranfield MK43 0AL, UK; vinod.kumar@cranfield.ac.uk

**Keywords:** starch, edible films, additives, active packaging

## Abstract

At present, people more actively pursuing biodegradable-based food packaging to lower the environmental problems of plastic-based packaging. Starch could become a promising alternative to plastic because of its properties (easily available, nontoxic, tasteless, biodegradable, ecofriendly, and edible). This review article is focused mainly on the impact of the properties of starch-based biodegradable films, such as their thickness, morphology, and optical, water-barrier, mechanical, oxygen-barrier, antioxidant, and antimicrobial properties, after the incorporation of additives, and how such films fulfill the demands of the manufacturing of biodegradable and edible food-based film with preferable performance. The incorporation of additives in starch-based films is largely explained by its functioning as a filler, as shown via a reduction in water and oxygen permeability, increased thickness, and better mechanical properties. Additives also showed antimicrobial and antioxidant properties in the films/coatings, which would positively impact the shelf life of coated or wrapped food material.

## 1. Introduction

In past decades, edible coatings received a great deal of attention as a promising approach to preserving fresh food products by reducing water loss, controlling respiration, improving glossiness, and inhibiting microbial growth during postharvest storage. The synthesis of common, reliable, and all-purpose coating solutions is a challenge for researchers because of the huge mechanical and physicochemical properties of the biopolymer. Edible coatings are generally derived from various biopolymers, e.g., starch, cellulose, protein, lipid, etc. [1].

Starch biopolymer has attracted great attention to its film-forming properties because of its low cost, abundance, and organoleptic (flavorless, tasteless, odorless), optical (transparent, colorless), and barrier properties (carbon dioxide and oxygen permeability) [2]. For film formation, these properties are ideal, but the water vapor permeability (WVP) and mechanical properties of starch biopolymer are low [3,4]. To ameliorate the WVP and mechanical properties of starch biopolymer-based films, researchers have been investigating the incorporation of cobiopolymers and other additives used during film formation [5,6]. At the same time, thermal treatment and postthermal modifications during storage may improve film formation or coating characteristics [4]. Furthermore, the effectiveness of pure matrix edible films is insufficient for practical applications, as the mechanical, barrier, antioxidant, and antibacterial characteristics of the film and coating are insufficient [7]. As a result, scientists have been experimenting in recent years to discover appropriate additives to enhance the film’s original properties while still providing additional functional properties. Plant extracts high in polyphenols are often used as film additives. These impart added functional qualities such as antioxidant and antibacterial properties in starch films and improve the films’ physical and mechanical characteristics [8]. Plant-based essential oils are another additive commonly used to impart strong antibacterial properties in starch films [9,10]. In addition, various nanoparticles, such as metal, silver, and inorganic nanoparticles (silica), have been studied to increase film efficiency [11].

According to current research, adding additives and other active ingredients to starch polymers could enhance their water barrier properties. Plasticizers improve the stability and strength of polymers and are utilized to form effective edible coatings and films [12]. Most starch-compatible plasticizers have been specified as polyols, e.g., mannitol and glycerol [13]. Modified starches (nanostarch particles) have significance because of their improved properties. They have become an emerging area for finding new possibilities in the applications of nanoparticles in starch film [13,14]. More recently, fatty acids; natural plant extracts such as lotus leaf extract [15], green tea extract [16], pitanga leaf extract [17], and pomegranate extract [18]; and organic compounds such as urea [19], sunflower oil [17], and essential oils have been used as additives and microbial inhibitors.

## 2. Starch-Based Edible Coatings/Films

Starch is a natural, biodegradable polymer that can be utilized to form beneficial food packaging substances. Researchers have studied it extensively because of its large quantity, low cost, edible nature, and biodegradability. Many raw agricultural food items, such as wheat, rice, corn, beans, and potatoes, contain starch, which is a form of natural biopolymer [18,20]. According to [21] more than 60% of cereal kernels contain starch, which is comparatively simple to isolate from the other components. The form, structure, size, and chemical composition of starch granules vary depending on the botanical source [22,23].

Amylose and amylopectin are the two major components found in starch, and other elements, such as lipids and proteins, can be found in trace quantities in starch granules. Amylose is a linear chain polymer with a molecular weight of 20 to 800 kg/mol made up of 1,4 anhydroglucose units. Many granular starches contain between 20 and 25% of this substance [21,24]. Amylopectin is a heavily branched-chain polymer with α-1,4 chains connected by α-1,6 glycosidic linkage per 25 to 30 glucose units and a high molecular weight (5000 to 30,000 kg/mol) [21,24,25]. The molecular and film-forming properties of amylose and amylopectin vary because of structural and molecular weight variations. Most starches are semicrystalline compounds, with crystallinity ranging from 15 to 45% based on the amylose (20–25%) and amylopectin (75–80%) [26,27]. The crystalline areas are produced by short-branched amylopectin chains, whereas the amorphous areas are produced by amylose and amylopectin branching chains [28]. The hydrogen bonds that keep the starch chains together prevent starch granules from dissolving in cold water. When starch is heated in water, its crystalline structure is disrupted because of the interaction of the hydroxyl groups present in amylose and amylopectin, which show partial solubilization [29]. Heating starch suspensions in extra water causes hydrogen bonding to develop, and an irreversible gelatinization reaction occurs at temperatures ranging from 65 to 90 °C, depending on the type of starch [30].

**Figure 1:**
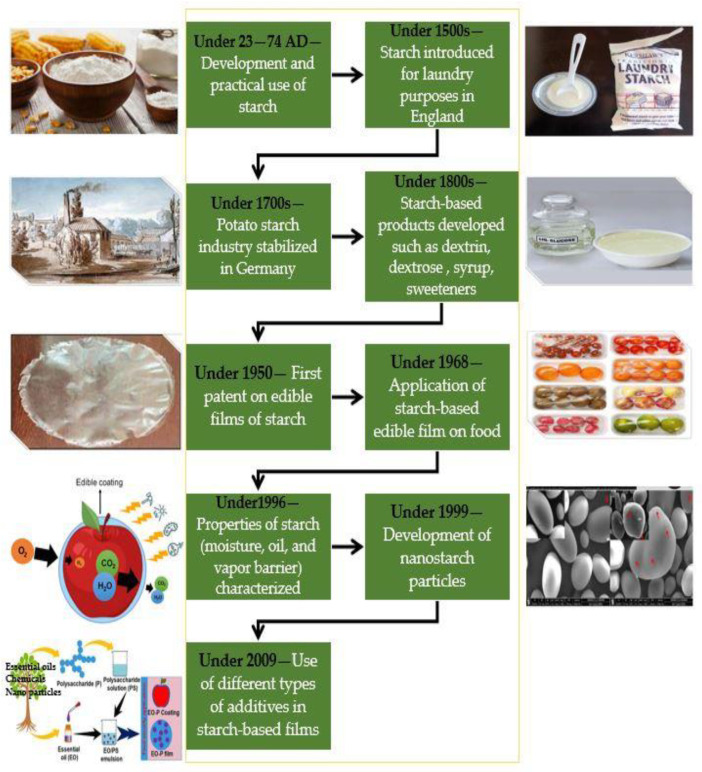
Timeline of starch and starch-based edible films/coatings.

Two methods are often used to make films from starch: the dry method and the wet method. Starch is heated above its glass transition temperature in the dry phase method, which uses thermoplastic properties by extrusion. The starch is then plasticized with low water content. The thermoplastic starch requires a plasticizer, particularly glycerol, to reduce the phase-transition temperature (i.e., the melting point) to be lower than the degradation temperature [31]. In the wet method, polymers are solubilized first, and then the film-forming solution is dried. The wet process is typically used to make edible pre-formed films or add coatings to food products by dipping, spraying, or brushing [26,32]. However, dry methods are more practical for film production [28]. Starch films have high oxygen barrier capabilities due to their highly ordered hydrogen-bonded network structure. The amylose and amylopectin form crystalline and noncrystalline portions in alternating layers [26]. As a result, increased crystallinity or higher amylopectin content in the sample improves the barrier properties. Compared with synthetic polymers, starch-based films have certain disadvantages, e.g., the mechanical properties are weak and the elongation ratio is low. However, the tensile strength is comparatively high. Although hydrophilic, starch films with greater crystalline content are sensitive to moisture and relative humidity in the atmosphere [26,33]. Water plays a significant role in the gelatinization and retrogradation (crystallization) of starch. Amylopectin crystalline regions can adopt several hydrated polymorphic forms; however, reports have differed on water migration during retrogradation [34]. The amorphous regions created by amylose are responsible for starch film’s poor mechanical properties, especially brittleness. To improve the film brittleness affected by intermolecular forces, plasticizers and additives must be added to improve flexibility and extensibility. Blending starch with other bio- or synthetic polymers, for example, polyvinyl alcohol (PVA), is another way to boost these properties while retaining biodegradability [20,35]. The history of starch-based films/coatings is shown in Figure 1.

## 3. Food Additives

A food additives was described as any compound that is intentionally added in the manufacturing, processing, treatment, preparation, packing, transport, and holding of food to perform a technological function according to the Codex Alimentarius (FAO/WHO, 2016), which is recognized as the international standard. The use of food additives enhances the finished product’s consistency and extends the shelf life of foods on store shelves. Additives should be used in a defined limit that does not negatively impact health or give an allergic reaction to the customer. By implementing the Codex Alimentarius, the FAO/WHO (2017) and the European Union (EU, 2017) defined the criteria under which the food industry can utilize food additives [36].

The major focus of this review is on the impact of various additives on the physical, water-barrier, biodegradability, mechanical, antioxidant, and antibacterial properties of edible starch-based films.

## 4. Functions of Essential Oils and Extracts as Additives

Various essential oils and extracts from plants have shown many beneficial effects in starch-based films and coatings (Table 1). Essential oils are composed mainly of ethers, phenols, terpenes, ketones, aldehydes, carbohydrates, and alcohols, which are responsible for their extraordinary biological properties [37]. Terpenes were assessed to be the most prevalent antimicrobial chemicals, accounting for 56.8% of the total, followed by polyphenols (phenolic acids, flavonoids), which accounted for 32.4% [38]. They are specifically used for improving antifungal and antimicrobial properties against both Gram-positive and Gram-negative bacteria and against many foodborne pathogens such as *Staphylococcus aureus*, *Campylobacter*, *Listeria monocytogenes*, *Salmonella* Typhimurium, and *Escherichia coli* by rupturing the plasma membrane and causing intracellular material to flow [39,40]. Despite the fact that little is known about the structure–function link of antibacterial phytochemicals, conjugation with sugar moieties at certain sites of the aromatic ring increases antibacterial ability [38,41]. Incorporations of essential oils into the films provided antimicrobial functions by release into the vapor phase where direct contact occurred between the films [42]. It also increased the antioxidant properties of films [43] and helped inhibit oxidation. Some studies have shown that film has low water vapor permeability and high crystallinity and hydrophilicity [44]. The development of hydrogen bonds between the hydroxyl groups of corn starch and essential oils with the addition of glycerol improves the firmness, thickness, and water vapor permeability of the film. Essential oils added to the surface of a starch film by the homogenization process were distributed uniformly over the surface, and because of the mixing of emulsifiers into the film-making process, the size of essential oil particles and the porosity of the surface decreased. This process also increased the physiological properties of the starch film [45]. The essential oil contains films/coatings that take more time to biodegrade in soil because of antimicrobial agents.

## 5. Function of Chemicals as Additives

The incorporation of chemicals into films showed the highest elongation at break, opacity, rigidity, and solubility [49] and increased physical and mechanical properties due to the higher intermolecular bonding among chemicals (potassium sorbate, etc.), polymers, and plasticizers [56]. Furthermore, potassium sorbate helped inhibit the growth of various foodborne pathogens, such as *Penicillium* spp., *Candida* spp., *Salmonella*, and *S. aureus* spp. [57]. The strong intermolecular interaction affects the chemical structure and molecular space in the network, resulting in reduced moisture adsorption and oxygen permeability with increased tensile strength and elongation ratio of the resulting starch-based film [48]. Chemicals reduced the mobility of the polymeric chains via cross linking reactions in the matrix. This directly positively affected the water and oxygen permeability and mechanical properties of the resulting starch-based film [58]. The reaction of water and another chemical compound to form two or more products involves the ionization of water molecules and usually the splitting of the other compound [59]. Some chemicals, such as nitrate, potassium sorbate, etc., have fine particles that fill the voids of starch-based films. This directly decreases the wrinkles in the resulting films, but because of the hydrophilic properties of these chemicals, it reduces the strength of the films [60]. Different effects of various chemicals on starch films are shown in Table 2.

## 6. Function of Pigments and Others as Additives

Pigments are generally rich in antioxidants and therefore used as additives to improvement in the properties of films [65]. They also improve the antimicrobial activity against coliforms and mesophilic aerobic bacteria and fungi [66]. The addition of a hydrophobic compound, such as carotenoid, in a hydrophilic matrix can modify the interaction between the chains of the polymer matrix, which decreases the polymer–polymer interaction and promotes the formation of discontinuities in the structure, reducing the tensile strength and elasticity of the films [67]. The lower permeability is due to the interaction of hydrogen from starch-based films and polyphenolic compounds, which reduces the availability of groups to form hydrogen bonds with water molecules and leads to films with lower affinity for water and gases (O_2_ and CO_2_) [60,68]. Prebiotics as additives improved nutritional characteristics, increased antioxidant activity, and hindered the penetration of water molecules [69]. Shahrampour et al. [70] found that the addition of L. plantarum cells to the film-forming solution improved the barrier performance of the film because of the hydrogen bond between the bacterial cells and the film-forming agent reducing the intermolecular distance [70]. Increased compactness of a starch film directly influences the mechanical properties of the film [69]. Mango and acerola (Barbados cherry) pulp improved the nutritional characteristics of films [65]. Details of various pigments and other additives used in film preparation are mentioned in Table 3.

## 7. Effect of Incorporation of Additives on Various Properties of Starch-Based Films

The impact of various types of additives on the physical, water barrier, biodegradability, mechanical, antioxidant, and antibacterial properties of edible starch films are discussed in the following section.

### 7.1. Thickness

The thickness of edible starch films influences their physical and mechanical properties. The thickness of the film has a strong relationship with the permeability properties of the film [7,75]. The addition of additives has led to minor increases in the thickness of edible starch films.

The incorporation of 6% cotton CNC (cellulose nanocrystal) in a corn starch film improved the thickness of the film by 22% as compared with the control film [76]. Because of the CNC incorporation, film thickness increased, with high solid content in the film and changes to the original structure of the film matrix [77]. Qin et al. [78] observed that film incorporated with *Lycium ruthenicum* extract (LRE) was significantly thicker that with added betacyanin-rich red pitaya peel extract (RPPE), and the film with added RPPE showed a denser inner structure [65]. The addition of GAL (*Garcinia atroviridis* leaf) extract to the cassava starch film had no massive impact on the film thickness because of the small amount of extract used in the film [79]. Chakravartula et al. [46] reported no changes in thickness when adding pitanga leaf extract in cassava and chitosan films. The thickness values obtained from control banana starch films and a film containing microparticles with LA/OA (lauric acid and oleic acid)-incorporated SLM (solid lipid microparticles) varied from 0.088 to 0.099 mm [64]. A high concentration of *Cordia verbenacea* extract combined with a pregelatinized starch film resulted in the desired flavonoid content, even though the thickness of the films was 0.060 mm. Similar changes were observed in the moisture present in the films [50]. Film thickness grew 24% higher and ranged from 0.164 to 0.212 mm compared with the control cassava starch film using different concentrations of two compounds (green tea extract and palm oil) [16]. The banana peel film had the highest thickness, which was significantly different from the others because of the addition of a higher concentration of *Eriobotrya japonica Lindl.* leaf the extract to the film solution (4% *w*/*w*), which increased the solids content in the film [80]. The incorporation of different concentrations of lycopene in the cassava starch film matrix increased the solid content of the film, which improved the thickness of the film [68]. Incorporation with crystalline nanocellulose (CNC) from cotton linters was also performed. On the surface of CNC, two bacteriocins were immobilized from broth cultures of *E. faecium* and *P. acidilactici* and used in the corn starch film. The resulting films showed thicknesses ranging from 180 to 200 μm, with no significant differences between them [72].

### 7.2. Morphology

Film qualities such as water vapor permeability, mechanical properties, and optical properties are all influenced by the microstructure of the film [81]. Sodium bisulfite (NaHSO_4_) and glycerol added to corn starch can damage the internal crystal structure of starch; affect its binding capacity and thermodynamic properties; and enhance its gelatinization [82,83]. The development of hydrogen bonds between the hydroxyl groups of corn starch and polyvinyl alcohol (PVA) with the addition of glyoxal improves the firmness of the film. Similarly, Mittal et al. [62] reported that urea–formaldehyde cross-linked PVA/starch films had fewer surface cracks and starch particles in SEM images [45]. Compared with the control starch film, the glyoxal cross-linked starch film had an even cross-section. CNE (carvacrol nano emulsion) added on the surface of a corn starch film by the homogenization process was distributed uniformly over the surface. With emulsifiers mixed into the film-making process, the size of essential oil particles and the porosity of the surface decreased. The addition of CNE into corn starch film did not affect the polymer crystallization peak of the matrix [45]. SEM imaging showed that when GAL extract (2 to 5%) was added to the tapioca starch film, the film surface turned less smooth and more uneven than that of the control film. GAL extract between 3 and 5% in the film led to a reduction in peak intensity in X-ray diffraction (XRD) analysis, which showed the amorphous structure of the film.

In comparison, control films and starch/gelatin films with 1% GAL extract showed a similar XRD pattern, indicating a more crystalline structure [79]. There were minimum changes in the structure of the potato starch films due to AOE (antioxidant extract from rice straw) incorporation. The occurrence of a uniformly fractured layer at the film surface in some films indicated that crystallization evolved in the film because of the increased molecular mobility caused by water vapor transfer to the film outer surface [54]. The addition of cellulose fiber and antioxidant extracts in potato starch–glycerol film molecules shifted slightly toward shorter amylose chains but did not affect the distribution of amylopectin [54]. However, the branch chain size distribution of amylopectin moved towards a higher chain length after citric acid was incorporated into the starch films, thus resulting in a monomodal distribution of chains [17,84]. An unripe banana starch film containing SLM in which LA/OA was incorporated showed some cracks in the film structure. The SLM caused irregularities on the outer surface of the starch film and increased the starch matrix gap [64]. In ungelatinized starch/polyvinyl alcohol blend films, a droplet phase was observed by SEM analysis, while in gelatinized starch/PVA, a laminated phase was observed, as the addition of glycerol/urea increased homogeneity of the blend films [19]. Fillers of crystalline nanocellulose (CNC), made from cotton linters, and bacteriocins immobilized in crystalline nanocellulose were also incorporated into the corn starch film matrices. The control film was smooth, whereas the CNC-incorporated film had less surface roughness under SEM, with a homogeneous and even distribution of filler components into the starch matrix in all micrographs [72].

### 7.3. Optical Properties

Optical properties are important in determining the capability of edible starch films to be utilized in food. These properties protect food against light exposure, particularly UV radiation-caused food deterioration. The light transmittance of a film in the range of 200–800 nm can be used to determine its transparency. Furthermore, studies have determined that the transparency value of the film is defined as the absorbance of the film at 600 nm divided by the thickness, which is referred to as “opacity” by most researchers [85,86].

When LRE and RPPE were added to cassava starch film, different colors (pink and purple) appeared, which were imparted by varied anthocyanin and betacyanin content. Other studies reported similar colors in films rich in anthocyanins or betacyanins [87]. The UV light transmittance of the films was effectively reduced by adding LRE and RPPE [78]. The opacity of tapioca starch films was significantly improved when 3–5% GAL extract was added. This could be related to the light scattering impact, through which the addition of extracts changed optical characteristics varying on the concentration used, which is essential for film transparency [79]. The films became increasingly transparent at longer wavelengths, with internal transmittance varying from 40 to 60% [54]. The incorporation of citric acid increased the transmittance of the potato starch films (with Ti of 85.8%), and higher homogeneity was indicated by higher Ti (internal transmittance) values. The optical characteristics were not affected by the addition of cellulose fibers [17]. The luminosity and opacity of the banana starch film decreased and increased, respectively, when nonencapsulated ascorbic acid was incorporated. The film had microparticles with lauric acid and oleic acid in a ratio of 80/20; when added with SLM-encapsulated ascorbic acid, luminosity decreased, and opacity increased. When the SLM was added to banana starch films, they grew darker and not so transparent [64]. Similarly, brown banana peel flour made the films darker and opaquer than other starch films. The addition of ascorbic acid to films generally changes their color [64]. A corn starch/SDS (sodium-dodecyl-sulfate) film matrix was more rigid, with stronger interactions between molecules due to the higher consistency and fewer vacant areas in the matrix, which was opaquer and inhibited light passage. In the case of starch/MEL (mannosylerythritol lipids) films, the biosurfactant behaved as a light-blocking agent by physically filling the gaps between the corn starch polymers [88]. Córdoba et al. [89] found that adding yerba mate extract decreased light transmission substantially. The addition of essential oil increased the opacity of corn starch films, and it was suggested that the essential oil drops caused refraction and dispersion of light. Despite the increased opacity, the mixing of CNE (carvacrol nanoemulsions) improved the UV-blocking capacity of the film, and the physical appearance of the film was not affected [90]. CNE increased refractive index, and larger specific surface area reduced film transparency. CAR (carvacrol) also provides excellent UV protection due to its abundant phenolic hydroxyl groups [45]. Cassava starch composite film transmittance decreased significantly under visible light after the addition of two probiotics, LPL and PPE (*Pedocococcus pentosaceus* and *Lactobacillus plantarum*, respectively). In CS/CMC/PPE-2%, transmittance was increased by 6.28% over that of CS/CMC (carboxymethyl cellulose) film [73]. Control films had the highest rate of transmission of corn starch films. Cotton linters were used to make films that contained crystalline nanocellulose (CNC). Bacteriocins were added to the surface of CNC and used to strengthen the corn starch film, which was isolated from broth cultures of *E. faecium* and *P. acidilactici*. When compared with control films, bacteriocin-immobilized crystalline nanocellulose (BIN) reduced the transmission rate by 14–16%, and the incorporation of BIN in films decreased the transmission rate by 27–29% [72].

### 7.4. Water Barrier Properties

The permeability of a film is influenced by its chemical structure, degree of crystallinity, cross-linking degree, polarity, density, polymerization, and molecular weight, with extra plasticizers [7,10]. Moreover, the hydrophilicity and hydrophobicity of the film composition are largely determined by the barrier to water vapor. Compared with the control sample, a cassava starch matrix with added glycerol showed a fiftyfold decrease in water vapor permeability (WVP) [16]. Betacyanins formed strong hydrogen bonds in the film matrix, which may have lowered the hydrophilicity of the starch films, increasing their water vapor barrier [68]. Adding glycerol, which interacts with starch chains, to the starch film increases free volume in the film network. It enhances molecular mobility, making hydroxyl groups more accessible to hydrogen bonding interactions with water. When phenolic compounds are replaced with glycerol, this lowers the water attraction in films because of an intermolecular reaction between glycerol and hydroxyl groups of starch chains [54]. According to one report, incorporating citric acid into potato starch films increased water vapor permeability [91,92]. The mixing of fatty acids to cassava starch films influenced their water barrier characteristics. Fatty acid absorption into a polymeric film, because of the effect of lauric acid, effectively lowered WVP at relative humidity from 33% to 64% [64]. The type of surfactant used also influences the WVP of cassava starch films. MEL (mannosylerythritol lipids) increased WVP compared with the control film, while SDS (sodium dodecyl sulfate) decreased WVP [46]. Kong et al. [45] observed that the pure corn starch films had high permeability to water vapor due to their hydrophilicity [45]. Lower WVP results were when higher concentrations (1.5 wt%) of citric and tartaric acids were used, indicating that the polymeric chain’s mobility was reduced because of cross-linking reactions, making water diffusion through the film matrix more challenging [58]. A higher concentration of malic acid resulted in films with increased water vapor permeability, possibly because of the difficulties of promoting transesterification reactions compared with the other acids evaluated and possibly because it contributed to the plasticizing impact. The hydrophobic nature of lycopene decreased its interface with water molecules and led to matrix discontinuities, resulting in a lower WVP and film elongation in cassava starch [68].

### 7.5. Biodegradability

Biodegradability studies simulates the breakdown conditions that occur in the environment because of soil microflora activity [93]. Biodegradability studies of starch films in composite soil imitate the degradation process in natural environments, where soil microflora contains bacteria, fungi, protozoa, and actinomycetes that act together in the biodegradability process [68,94].

During the first 15 days, the starch–chitosan–nanoclay bionanocomposite films degraded quickly. They then degraded slowly and regularly for the next 60 days. The corn starch–chitosan–nanoclay (control) film decomposed quicker than the other bionanocomposite films, as it lacked an antibacterial component [48]. In an analysis, weight reduction occurred; this might have been attributable to enzymes and microorganisms and the water added to the soil solutions containing the soluble components of the starch film [80]. Degradation of CS (cassava starch)/CMC (carboxymethylcellulose)-based composite films with probiotics showed a significant mass loss, because both CS and CMC are hydrophilic degradation materials [69]. The cassava starch films containing 5% nonencapsulated lycopene showed quick biodegradability and enhanced biodegradation rate, with a mass loss of 35 to 38%, indicating them as a good source against nondecomposable polymers and poor disposal of these materials [68]. Jaramillo et al. [95] observed similar results after treatment with yerba mate extract and cassava starch. The material was biodegraded in 12 days, highlighting the need for biodegradable packaging and alternative nonbiodegradable polymers [94]. Biodegradable films made of starch and glycerol, which are hydrophilic substances because of their enhanced water absorption, might lose a lot of mass during the biodegradation process [96,97]. Corn starch films containing crystalline nanocellulose (CNC) made from cotton linters showed the most deterioration, and films containing bacteriocins exhibited the least deterioration [72]. Biodegradation behaviors of modified starch-based films depended on the hygroscopicity of the polymer matrices [98].

### 7.6. Mechanical Properties

The stress range of edible starch films is directly determined by their mechanical characteristics in the actual use of food packing. The elongation at break (EB) and tensile strength (TS) of a film are the common factors that represent its maximum stress strength and flexibility [99]. The interaction force and internal structure of the film matrix are mainly responsible for the mechanical characteristics of films.

Bitencourt et al. [100] studied that blending cassava starch film with PVA enhanced the TS and EB significantly. In addition, Qin et al. [78] observed that when a percentage of RPPE was added to the film formation, TS increased because of the formation of hydrogen bonds between the extract and the film matrix, which reduced the intermolecular forces between adjacent macromolecules and enhanced the mobility of polymeric chains [68]. Corn starch films composed of chitosan/nanoclay/sorbitol/GFSE showed high tensile strength due to the presence of chitosan hydroxyl and amino (–NH 2) as a functional group [48]. Adding GAL extracts to tapioca starch films enhanced their mechanical characteristics, as TS and EB increased considerably [79]. The addition of AOE to potato starch glycerol films increased their TS but decreased their flexibility. EB was reduced from 26% to 14% when the antioxidant extract was added to the film, but Young’s modulus (rigidity) and tensile strength were enhanced [54]. Potato starch film with citric acid had higher TS than starch glycerol films but showed lesser extensibility. Chemical cross-links between citric and starch chains acid may have created a stronger network, but some minor cracks were observed in the film. Films containing cellulose fibers, citric acid, and antioxidant extract had favorable properties regarding extensibility and tensile strength [92]. A banana starch film with nonencapsulated ascorbic acid had greater EB than a control film. In the starch film medium, ascorbic acid as an additive behaved as a plasticizer that lowered the film TS and Young’s modulus [64,101]. When 10% CNE was mixed into the film matrix, the Young’s modulus value increased to 11 MPa, higher than that of the control film (5.6 MPa). However, when the concentration of CNE increased from 10% to 25%, the values of Young’s modulus dropped from 11 MPa to 6.1 MPa. Furthermore, CNE films showed stronger elongation at break than control films, increasing from 23 to 133% [45]. Pregelatinized starch films with higher concentration of cordial verbenacea extract showed lower TS and higher elongation [50].

### 7.7. Antioxidant and Antimicrobial Properties

Edible starch films have significant antioxidant and antibacterial effects and prevent packaged food from deteriorating. They act as an important barrier to the surface food and effectively inhibit the growth of spoiled bacteria in packaged foods [8].

Free radicals can cause food to degrade and nutritional content to deteriorate. Extracts consisting of phenolic hydroxyl groups, anthocyanins, and betacyanins, which have H-donating capacity, have shown high free radical scavenging activity in films and coatings [102]. Antioxidant-containing films can convert purple DPPH radicals to yellow, since starch film lacks antioxidant components [103].

LRE and RPPE have considerable antioxidant and antimicrobial properties, which helped in improving the properties of casava starch film, because anthocyanin phenolic hydroxyl groups might bind to free radicals and provide phenolic hydrogen atoms [104]. Notably, because of the different structures of anthocyanins and betacyanins, the antioxidant activity of RPPE film was much greater than that of LRE film. Because betacyanins damaged the structure and membrane permeability of bacteria, RPPE film exhibited a high antibacterial impact, resulting in microbial death [105]. Lozano-Navarro et al. [106] reported that the addition of RPPE to chitosan–starch films improved antibacterial activity against *E. coli* and fungi (*Penicillium notatum*, *Aspergillus fumigatus*, and *Aspergillus niger*) [104]. The antibacterial activity of RPPE film was stronger against Gram-positive bacteria than against Gram-negative bacteria [107]. The presence of polyphenols in GAL extract significantly increased total phenolic content values and allowed it to act as a natural antioxidant. The phenolic ring, which can delocalize unpaired electrons and transfer hydrogen from the hydroxyl groups, contributed to the antioxidant activity of the phenolic groups [108]. This could be a preliminary indication of the antioxidant activity of tapioca starch film [79]. Aqueous rice straw extract also led to improvement in the antioxidant activity of potato starch films. Antioxidant extract-containing films had increased action against DPPH radicals, which was linked to the amount of antioxidant extract in the films [54]. When antioxidant extract, citric acid, and cellulose fibers were added to potato starch film, the film showed higher free radical scavenging activity than the control film. In combination with the phenolic ingredient, citric acid significantly improved the antioxidant capabilities of the films and protected food from oxidation [17]. A considerable rise in DPPH free radical scavenging activity was seen with the addition of CAR (carvacrol); the maximum activity of the film was 81% (carvacrol and corn starch), and the control corn starch film had restricted antioxidant activity at 32% [45]. The CAR (cavacolphenolic) hydroxyl group could be used as a peroxy radical donor throughout oxidation, avoiding the development of peroxy hydroxyl groups and the chain reaction of lipid peroxidation and thus defending the lipid matrix from oxidation, decreasing oxidative damage, and increasing the shelf life of film [109,110]. Cordial verbenacea extract presented 90.5% inhibition of the COX-2 enzyme, which demonstrated the antiinflammatory potential of the plant [46]. Films with cordial verbenacea extract had a high COX-2 enzyme inhibitory capacity. Increasing the flavonoid concentration in the pregelatinized starch films, and thus the herb extract’s concentration, promoted higher antiinflammatory ability [50]. Corn starch films seemed to inhibit both test organisms in antimicrobial tests (*S. aureus*, *E. coli*). Films containing crystalline nanocellulose (CNC) made from cotton linters had the lowest activity against microorganisms, whereas those containing bacteriocins had the maximum activity. BIN in the films reduced bacterial activity against microorganisms by up to 17% as compared with films without bacteriocins, whereas it increased significantly by three times as compared with films with CNC [72]. The addition of the additives pitanga (*Eugenia uniflora* L.) leaf extract (PE) and natamycin (NA) improved radical scavenging activity in cassava starch films from 7.68% to 86.20%. Pitanga extract contains phenolic and pigment compounds that may have contributed to the film’s increased antioxidant capability. When compared with films without additions, natamycin alone improved antioxidant activity because of its compositional impact [54].

### 7.8. Oxygen Barrier Properties

Starch films have good oxygen barrier characteristics, because amylose and amylopectin have highly ordered hydrogen-bonded network structures containing crystalline and noncrystalline regions in alternating layers, leading to more permeability with the mixing of other additives [111,112].

The plasticizing impact of water content and glycerol-influenced oxygen permeability showed greater values with more water content and glycerol. Glycerol increased the space within and between starch components while decreasing interchain hydrogen bonding, allowing starch chains to glide and the substance to elongate [111,113]. The oxygen permeability was reduced when AOE was added to the starch glycerol film. In the film, interaction between the starch hydroxyl group and phenolic compounds led to increased tensile strength and oxygen permeability and reduced film permeability. The addition of starch nanoparticles generally reduces the oxygen permeability of various polymer matrices [114]. Starch films with sufficient antioxidant extract showed the maximum increase in oxygen barrier qualities compared with the control starch–glycerol films [54].

## 8. Food Applications

Consumer demand for healthy and safe meals has prompted scientists to look for more natural alternatives to better food quality and safety. Since edible components have been accepted as additives by the European Commission (EC) and the Food and Drug Administration (FDA) and are now more likely to be used in and on food items rather than as synthetic preservatives [115] by incorporating active ingredients such as essential oils in coating and film development for preservation of fruits, vegetables, and meat. These packaging methods might have a bright future, as they prolong the shelf life of these items by reducing moisture loss and delaying microbial degradation and lipid oxidation [116,117].

The addition of *Citrus limon, Rosmarinus officinalis*, and *Vitis vinife*ra essential oils into packing materials reduced the formation of biogenic amines in fresh fruit [118]. de Aquino et al. [119] studied the impacts of chitosan and cassava starch coatings with essential oil of *Lippia gracilis* Schauer to preserve guavas *(Psidium guajava* L.) after 10 days of storage at 25 °C. In this study, blends of chitosan, cassava starch, and essential oils were shown to be more efficient at inhibiting the development of most bacteria [116]. The overall number of mesophilic aerobic bacteria, as well as the counts of yeasts and fungus, were significantly reduced in the film-coated fruits on the 10th day of storage, and the total soluble solid content of the coated fruits was not much affected.

Evangelho et al. [117] studied the physicochemical and qualitative characteristics of mango using starch films containing thyme EO (essential oil) microcapsules. Compared with films without EO, starch films with added EO had a higher preservation effect. Starch films with EO applied on mangoes led to a shelf life of 10 days at room temperature (25 °C), indicating the film’s potential for mango preservation.

Issa et al. [118] studied the use of starch films activated with thyme essential oil in spinach leaves. The populations of *Escherichia coli* and *Salmonella typhimurium* decreased with the addition of EO on fresh spinach leaves.

Radha et al. [119] studied the application of maize starch films with spice oils (cinnamon and clove) to preserve raw beef and showed a reduced microbial population during refrigerated storage at between 3 and 5 °C for 15 days, as well as enhanced color stability during the storage period. This study suggests that this substance might act as an extra barrier in meat products to reduce microbial degradation and stop lipid oxidation.

Caetano et al. [120] showed that the antioxidant and antibacterial activities of cassava starch films with essential oregano oil were effective and that such films showed protection against ground beef oxidation up to the third day of storage. The use of cassava starch films mixed with lemongrass essential oil resulted in a lower microbial meat count throughout the storage [116,121].

Amiri et al. [122] studied the sensory and physiochemical properties of ground beef hamburgers by applying corn starch films with EO from cinnamaldehyde and *Zataria multiflora*. Compared with the film without EO, the film with EO showed positive results in sensory physiochemical parameters after storage [123].

## 9. Conclusions

The major goal of this review was to present the recent and innovative work in the development of starch-based films with additives such as essential oils, chemical agents, pigments, and other edible compounds. Starch types, time and temperature during film formation, cobiopolymers, plasticizers, and storage conditions affect the properties of the starch film. Additives are very easy to obtain and have shown highly antibacterial and antioxidant properties, which can improve the shelf life of food. However, the drawbacks of lower water vapor barrier, mechanical strength, and thermal stability remain the main challenges that must be solved before starch-based films can compete in the food packaging industry. Simultaneous incorporation of additives is a viable strategy partially compensating for the aforementioned loss of characteristics. In view of the composite films that have been developed, the compatibility of additives with the biopolymer matrix can also help to improve the results in future studies. Another attractive feature to consider is the absence of comprehensive studies on the stability of additives in terms of their antioxidant, antibacterial, and other properties in film. Furthermore, standardization of the additives potentially impacting material consistency in film manufacture needs to be investigated.

## Figures and Tables

**Table 1:** Effects of essential oil/extract on starch-based films/coatings.

Sources of Starch	Additives	Amounts Incorporated	Plasticizers Used	Key Features of Developed Films	Reference
Cassava starch	Green tea extract (2.5, 5.0 and 7.5%) and palm oil colorant (0.01, 0.05 and 1.00%)	2.5–7.5% 0.01–1.0%	Glycerol	▪ The resultant coatings had improved mechanical, water-vapor barrier, and antioxidant characteristics; reduced the peroxide index in packaged butter and could provide oxidative protection.	[16]
Corn starch and banana peel flour	*Eriobotrya japonica* leaf extract	4%	Glycerol	▪ Increased elongation and deformation rates; increased total phenolic content, solubility, and antioxidant activity; decreased the elastic modulus, water vapor permeability, and mechanical resistances.	[17]
Corn starch	Zanthoxylum bungeanum essential oil	0.5%, 1.0%, and 2.0% *v*/*v*	Glycerol	▪ Films showed lower tensile strength but high EB; water-vapor permeability was lowered, opacity was enhanced, and gloss was reduced; improved antibacterial activities against Gram-negative and Gram-positive bacteria.	[40]
Chitosan and sugar palm starch	Extra virgin olive oil	1%, 2%, and 5%	Glycerol	▪ Thermal stability and antioxidant activity were both improved; surface roughness was reduced; the film showed the highest EB and tensile strength.	[43]
Corn starch	Cinnamomum camphora, cardamom, and cinnamon oil	2% and 5%	Glycerol	▪ Excellent mechanical properties; low crystallinity and thermal stability, as well as low melting temperatures; addition resulted in a slight drop in WVP values.	[44]
Corn starch	Carvacrol nanoemulsions (CNE)	5 mL, 10 mL, 15 mL, and 20 mL	Glycerol	▪ Enhanced water-vapor and UV-light barrier properties and mechanical strength; showed high antifungal and antioxidant activity.	[45]
Chitosan and cassava starch	Pitanga (*Eugenia uniflora L*.) leaf extract and natamycin	1 g and 2.25 g	Glycerol	▪ Increased antioxidant and antifungal activity; increased opacity enhanced the light-barrier characteristics of the film.	[46]
Cassava starch	Mannosylerythritol lipid-B	20 g	Glycerol	▪ Films had the highest flexibility and superhydrophobicity; showed antimicrobial activity.	[47]
Corn starch	Grapefruit seed extract (GFSE)	0.5 g and 1.5 g	Glycerol and sorbitol	▪ Films had high crystallinity; low WVP and hydrophilicity; and improved mechanical properties.	[48]
Corn starch	Carvacrol essential oil (EO) and montmorillonite (MMT)	4.5 wt%, 9 wt%, and 15 wt%	Glycerol	▪ Decreased thermal stability and crystallinity; increased antimicrobial activity against *E. coli*.	[49]
Pregelatinized starch	*Cordia verbenacea (erva baleeira*)	0.25 mg, 0.50 mg, and 0.75 mg	Sorbitol	▪ Enhanced heterogeneity and elongation; reduced the tensile strength mucoadhesive force of the films; in storage, maintained flavonoid content and antiinflammatory activity.	[50]
Cassava starch	Yerba mate (Ilex paraguariensis) leaf extract	10 wt% and 20 wt%	Glycerol	▪ The density of fractured starch granules was reduced; lowered water-vapor permeability; changed color in the films.	[51]
Corn starch and chitosan	*Thymus kotschyanus* essential oil and pomegranate peel extract	0.5%, 1%, and 2% (*w*/*w*)	Glycerol	▪ Inhibited bacterial counts and lipid oxidation; increased the physicomechanical properties of composite films.	[52]
Corn/octenylsuccinated starch (C/OS)	Soybean oil (SO)	0.5%, 1.0%, 1.5%, and 2.0% *w*/*w*	Glycerol	▪ Improved surface hydrophobicity and water-barrier property; increased tensile strength.	[53]
Potato starch	Rice straw waste	2 g, 3 g, and 4 g	Glycerol	▪ The film turned more brittle with increasing amounts of extract; increased oxygen barrier properties; produced at a low cost.	[54]
Tapioca starch	*Garcinia atroviridis* leaves	1%, 3%, and 5%	Glycerol	▪ Antioxidant properties improved; reduced water solubility; increased mechanical properties (tensile strength and elongation).	[55]

**Table 2:** Different chemicals and their effects on starch-based films/coatings.

Sources of Starch	Additives	Amounts Incorporated	Plasticizers Used	Key Features of Developed Films	Reference
Cassava starch	Sodium nitrite	1%, 2%, and 5%	Glycerol	▪ Enhanced mechanical properties and clarity in blend films; increased hydrophilicity and WVP due to nitrite but reduced oxygen permeability; reduced microbial growth, lipid oxidation, and metmyoglobin formation.	[31]
Cassava starch	Sodium-dodecyl-sulphate	20 g	Glycerol	▪ The film showed the highest elongation at break, rigidity, opacity, rigidity, and solubility in water; resulted in the lowest hydrophilicity and water vapor permeability.	[46]
Corn starch	Potassium sorbate (KS)	0.2 g and 1.5 g	Glycerol and sorbitol	▪ The film showed better physical, mechanical, and thermal properties.	[48]
Maize starch chitosan	Polyvinyl alcohol (PVA)	0–40 wt%	Glycerol	▪ Transparency of the film increased; mechanical properties and biodegradability improved; UV-protective properties increased.	[55]
Cassava starch	Poly (butylene adipate-co-terephthalate) (40, 60 g), coconut nanocellulose (0.55 g), annatto (0.5, 1.0 g), and citric acid (1.0 g)	40–60 g 0.55 g 0.5 g 1.0 g	Glycerol	▪ Increased physical and mechanical properties; reduced water-barrier property; increased stiffness of the films.	[56]
Corn starch	Potassium sorbate	0.1–0.5%	Glycerol	▪ More yellowness and less transparency than films without potassium sorbate; maintained a high antimicrobial concentration on product surface; inhibited *Penicillium* spp., *Candida* spp., *Salmonella*, and *S. aureus* spp. growth.	[57]
Thermoplastic starch with polybutylene adipate terephthalate	Sodium nitrite	1–5%	Glycerol	▪ Provided antimicrobial capacity, redness enhancement, and improved oxygen-barrier properties.	[61]
Corn starch	Urea	16.8 wt%	Glycerol	▪ Increased homogeneity of the blend films; decreased melting temperature.	[62]
Corn starch	Deep eutectic solvents (DES) (urea–choline chloride and glycerol–choline chloride)	2:1	Glycerol	▪ Reduced water sensitivity and recrystallization; compatibilization with a hydrophobic phase was obtained.	[63]
Banana starch	Lauric acid, oleic acid, and ascorbic acid	0.9%	Glycerol	▪ Lower water-vapor permeability; higher opacity and lower luminosity; reduced light transmittance values; elongation at break was lower, but tensile strength was higher; the rigidity of the films improved.	[64]

**Table 3:** Different pigments and other additives and their effects on starch-based films/coatings.

Sources of Starch	Additives	Amounts Incorporated	Plasticizers Used	Key Features of Developed Films	Reference
Cassava Starch	Acerola and mango pulps	0–20%	—	▪ Observed total carotenoid, total polyphenol, and vitamin C; increased antioxidant properties.	[65]
Rice starch/ Chitosan	Anthocyanins, betalains, resveratrol, thymol, and carvacrol	5%	Glycerol	▪ Showed natural antioxidants in small amounts; improved thermal and antimicrobial properties of the film; reduced fungi and mesophilic aerobic and coliforms bacteria; improved thermal and physical characteristics.	[66]
Cassava Starch	Lycopene or lycopene nanocapsules	2%, 5%, and 8% (*w*/*w*)	Glycerol	▪ Antioxidant activity was higher, light transmittance was reduced, opacity was greater, and protective oxidation was present; low elongation and tensile strength but improved solubility.	[68]
Cassava starch	Lactic acid bacteria (*Lactobacillus plantarum* and *Pedocococcus pentosaceus)*	0.5 g, 1 g, 1.5 g, and 2 g	Glycerol	▪ Reduced WVP and decreased light transmittance of the film; increased antioxidant activity; hindered the penetration of water molecules.	[69]
Cassava starch	Anthocyanins /betacyanins	3:1, 1:1, and 1:3	Polyvinyl alcohol (PVA)	▪ Increased the UV–visible light barrier property; showed high antimicrobial, antioxidant, and water-vapor barrier properties; ammonia-sensitive properties in film were observed.	[71]
Corn starch	Immobilized bacteriocin	1%	Glycerol	▪ Showed good antibacterial activity and biodegradability and better tensile strength; reduced optical transmission; the water solubility of starch films was reduced by 19 to 23%.	[72]
Cassava Starch	Papain	5–15%	—	▪ Improved tenderness of beef, with reduced Warner–Bratzler shear values and hardness.	[73]
Thermoplastic starch with polybutylene adipate terephthalate	Zinc oxide nanoparticles	1–5%	—	▪ Prevented microbial growth and increased lipid stability; extended shelf-life of packaged meat by more than 3 days.	[74]

## Data Availability

Not applicable.
